# High-Frequency Bipolar Coagulation Limits Epidural Fibrosis in Lumbar Microdiscectomy

**DOI:** 10.7759/cureus.45077

**Published:** 2023-09-12

**Authors:** Ahmed Yavuz, Haydar Gok, Kivanc Yangi, Suat Erol Celik, Gokhan Percinoglu, Kamber Goksu

**Affiliations:** 1 Neurosurgery, Prof. Dr. Cemil Taşçıoğlu City Hospital, Istanbul, TUR; 2 Neurological Surgery, Prof. Dr. Cemil Taşçıoğlu City Hospital, Istanbul, TUR; 3 Radiology, University of Health Sciences, Fatih Sultan Mehmet Training and Research Hospital, Istanbul, TUR

**Keywords:** dural injury, unplanned reoperation, bipolar coagulation, lumbar microdiscectomy, epidural fibrosis

## Abstract

Background and aim: We propose a vast study to examine the effect of high-frequency bipolar coagulation used in the operating room to prevent the development of epidural fibrosis after lumbar microdiscectomy.

Materials and methods: A total of 1004 participants were divided into two groups: no high-frequency bipolar coagulation (NC group) and high-frequency bipolar coagulation (C group). Postoperative epidural fibrosis, infection rates, reoperation status, and dural injury complications during the operation were recorded.

Results: Considering the epidural fibrosis rates of the two groups, epidural fibrosis was seen in 10.6% of the patients in the NC group. In contrast, it was seen in only 6.2% of the patients in the C group.

Conclusion: The complication of epidural fibrosis that develops after lumbar microsurgery operations both impairs patient comfort and brings with it the complications of reoperation. After performing hemostasis with bipolar, coagulating the annulus may effectively reduce epidural fibrosis and prevent reoperation.

## Introduction

Epidural fibrosis (EF) is a natural complication of the healing process after spinal surgeries. It is characterized by replacing epidural tissue with fibrotic tissue [1]. The process starts with the release of cytokines and the increased proliferation of fibroblasts [2]. EF takes approximately six weeks to six months to detect lumbar magnetic resonance imaging (MRI) postoperatively [3-5].

EF is a significant cause of failed back surgery (FBS), accounting for 10% to 24% of cases [6,7]. In the case of space-occupying lesions such as tumors [8], foreign bodies, or fibrotic tissues in the epidural area, patients may develop severe pain and neurological problems [9]. Fibrosis surrounds the periphery of the nerve root and can cause mechanical compression. Compressive tissue around the nerve causes lumbar disc herniation surgery inadequacy and causes patients to present with the same findings again [10].

High-frequency bipolar coagulation is a surgical instrument used for bleeding control in surgical operations. It also helps to coagulate epidural surrounding tissues. Coagulation of epidural tissues may cause tissue shrinkage and may even prevent the proliferation of epidural fibrosis [11].

We propose a vast study to examine the effect of high-frequency bipolar coagulation used in the operation room to prevent the development of epidural fibrosis after lumbar microdiscectomy.

## Materials and methods

This study included 1004 patients (474 females and 530 males) who underwent one-level unilateral microdiscectomy for lumbar disc herniation at our hospital between 2010 and 2019. The patients were assigned randomly. The participants were divided into two groups: no high-frequency bipolar coagulation (NC group) and high-frequency bipolar coagulation (C group). The same senior surgeon operated on all patients with the standard microdiscectomy procedure. The annulus fibrosis (a part of the intervertebral disc) was coagulated with high-frequency bipolar until no living tissues remained in the C group. Bipolar coagulation was avoided whenever possible in the NC group, and only surrounding hemorrhaging soft tissues were coagulated with a low frequency (20-30 Hz) if necessary. Non-steroidal anti-inflammatory drugs (NSAIDs) and a muscle relaxant were prescribed for one week, and steroids were not used. Patients were followed up on the 7th, 15th, 1st, 3rd, 6th, and 12th months. They have been followed up on for 12 months. During follow-up, patients were imaged with a contrast-enhanced MRI in cases of newly developed lower back or leg pain. Since the patients had no pain or complaints that did not develop during their 12-month follow-up, the surgical area was checked for fibrosis with contrast-enhanced lumbar MRI at the 12th-month follow-up. The presence of epidural fibrosis was checked. In control MRI images, it was checked whether there was a disc recurrence and fibrosis in the epidural area with the contrast enhancement of the epidural area. Epidural fibrosis was classified according to the extent of the enlarged area. Postoperative infection rates, reoperation status, and dural injury complications during the operation were recorded.

Only patients over the age of 18 who had one-level unilateral lumbar microdiscectomy surgery were included in the study. Patients who had previously undergone spinal surgery and were treated with epidural steroid drugs, required multilevel or bilateral disc surgery, or had any suspicion of instability were excluded from the study.

Statistical analysis

Data were analyzed using the Statistics Package for Social Science (SPSS 23.0, IBM, Armonk, NY, USA). Characteristics of patients, as n (percent) or mean ± standard deviation (SD) for categorical and continuous variables, were compared among treatment groups using Chi-square, Mann-Whitney U, and Kruskal-Wallis H tests. The Wilcoxon signed-rank test was used to compare the median of two dependent groups. The P-value was set at <0.001 for statistical significance.

## Results

**Figure 1:**
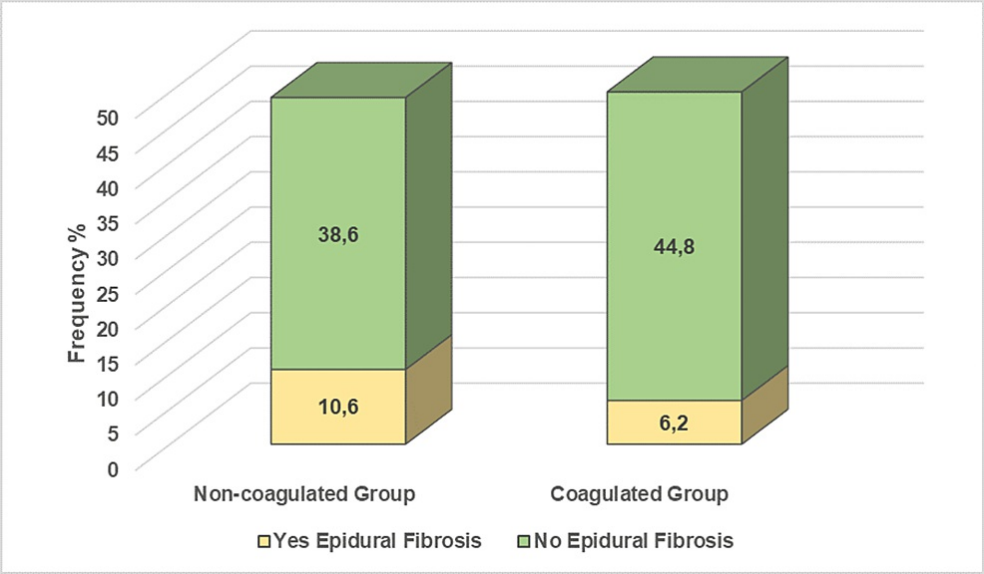
Comparison of the epidural fibrosis between non-coagulated group and coagulated group

The study included 1004 patients who underwent one-level unilateral lumbar microdiscectomy. The patients were divided into two groups: group NC (492) and group C (512). The mean age of the patients included in the study was 47, ranging between 25 and 75 years. 50.4% of the patients were men, and 49.6% were women. There were 492 patients in the NC group and 512 patients in the C group. The majority of the patients were operated on at the L5-S1 (48.9%) level. This is followed by L4-5 (26%), L3-4 (18.8%), L1-2 (3.7%), and L2-3 (2.7%). Considering the epidural fibrosis rates of the two groups, epidural fibrosis was seen in 10.6% of the patients in the NC group, while it was seen in only 6.2% of the patients in the C group (Figure 1).

**Figure 2:**
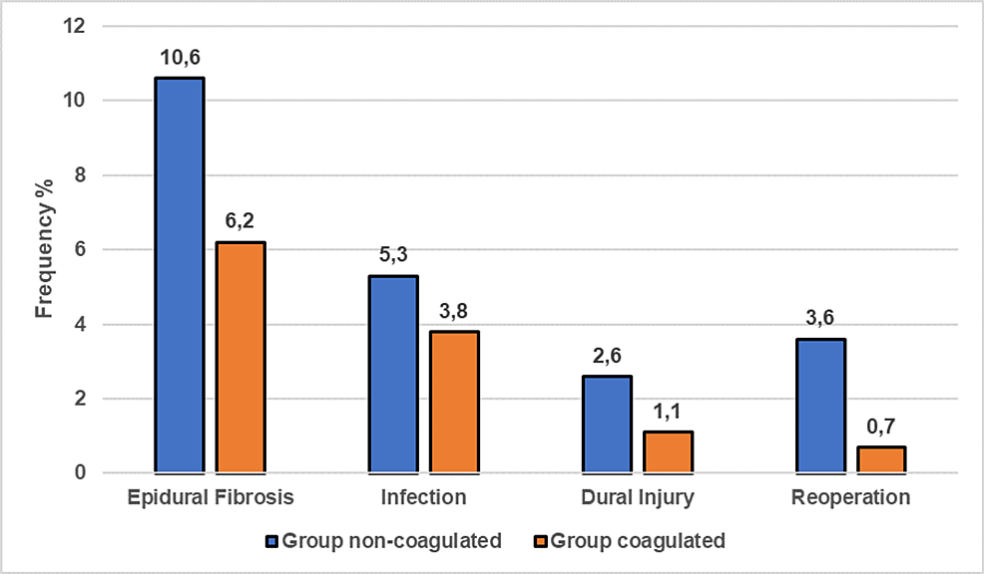
Comparison of non-coagulated group and coagulated group according to epidural fibrosis, infection, dural injury, reoperation rates

This difference between groups is statistically significant (p<0.001). When the patients had dural injuries during the operation, there was no statistical difference between the NC group (2.6%) and the C group (1.1%) (p = 0.008). Considering the postoperative infection rates of the patients, no statistically significant difference was observed between the NC group (5.3%) and the C group (3.8%) (p = 0.064). A statistically significant difference was observed between the NC group (3.6%) and the C group (0.7%) when the patients were reoperated (p<0.001) (Table 1) (Figure 2).

**Table 1:** Comparison of the results of the two groups with (C) and without bipolar coagulation (NC)

	Group NC (n=492)	Group C (n=512)	p-value
	n (%)/M(Q1 Q3)	n (%)/M(Q1 Q3)	
Female	245 (24.4)	253 (25.2)	0.903
Male	247 (24.6)	259 (25.8)	
Age	47 (25 72)	48 (20 75)	
Epidural fibrosis			
Yes	106 (10.6)	62 (6.2)	<0.001
No	386 (38.4)	450 (44.8)	
Infection			
Yes	53 (5.3)	38 (3.8)	0.064
No	439 (43.7)	474 (47.2)	
Dural injury			
Yes	26 (2.6)	11 (1.1)	0.008
No	466 (46.4)	501 (49.9)	
Reoperation			
Yes	36 (3.6)	7 (0.7)	<0.001
No	456 (45.4)	505 (50.3)	
Location			
L5-S1	228 (22.7)	263 (26.2)	0.509
L4-5	137 (13.6)	124 (12.4)	
L3-4	97 (9.7)	91 (9.1)	
L2-3	12 (1.2)	15 (1.5)	
L1-2	18 (1.8)	19 (1.9)	

## Discussion

Epidural fibrosis is a mass of tissue comprising a mixture of cellular elements, primarily fibroblasts and inflammatory cells. It is part of the usual healing process of the tissues surrounding the operated spine [6].

During tissue healing, polymorphonuclear neutrophils play a role in clearing pathogens and foreign particles. Still, they can also cause tissue injury by generating reactive oxygen species, promoting excessive migration of fibroblasts, and promoting collagen deposition. These processes can result in excessive scarring or adhesion formation [12]. EF formation typically occurs six weeks to six months postoperatively and is identified on MRI [3-5]. For this reason, we followed all the patients for 12 months and examined the epidural fibrosis status with contrast-enhanced lumbar MRI.

Adhesions that occur between the sheath of the nerve root and EF over time disrupt the elasticity and flexibility of the nerve tissue, which can lead to disruptions in axoplasmic transport, arterial circulation, and venous drainage within nerve fibers [13,14]. This level of EF can lead to severe neurologic insufficiency and clinical findings such as recurrent radicular pain or lower back pain [1,15]. Some complications, such as a dural tear, excessive bleeding, nerve damage, or infections, may occur because of reoperations [15,16].

Numerous clinical and experimental studies have been carried out to reduce or prevent the formation of EF using different surgical procedures and techniques, various biophysical barriers, and pharmacological agents [17-19]. However, only limited success has been achieved to date, and no adequate solution has been found to prevent it.

The first research on EF by Key and Ford revealed that the destruction of the annulus fibrosis might be to blame for the development of fibrous tissue and postoperative adhesions [20]. Even though surgical factors like using a lot of cotton pads, pulling out a lot of roots, bleeding, and root abnormalities have been linked to EF, fibroblast involvement, and EF are caused by injured paraspinal muscles [2,21,22]. This leads to symptomatic problems due to dura and nerve root irritation, entrapment, compression, tethering, and increased susceptibility to injury [7,23]. Furthermore, epidural adhesion causes various difficulties and complications in revision surgery [24]. Although Cinotti et al. did not find a correlation between the amount of fibrosis present and failed back syndrome [4], Mohi et al. found a strong correlation between pain intensity scores and the grade of EF [6]. Further, Ross et al. reported a significant association between the presence of extensive EF and the recurrence of radicular pain [1,25].

EF may occur as a result of the self-repairing process of the annulus. Thus, inhibiting or reducing the repair process of the annulus or directly repairing the defect may prevent the formation of fibrotic tissue. The smaller the amount of blood and blood products in the epidural area, the fewer cytokines will develop, which could reduce the risk of EF. This may be possible by disrupting the vascularity of the annulus and preventing blood and blood products from reaching this area [11].

Cetin et al. show the effects of bipolar coagulation on epidural fibrosis. This report accused blood and blood products of EF formation, and good hemostasis is required to reduce it [11]. However, Köksal et al. reported that using less bipolar cautery during laminectomy resulted in less EF in 14 rats, which did not support our findings. However, the methods used in their study differed from ours: we focused on explicit coagulation of the annulus fibrosus, whereas they coagulated the dura after performing laminectomy [26].

In our large-scale study, we focused on the bleeding areas and coagulated the annulus, which is the fibrous layer inside the intervertebral disc. We also found that radiological EF development was less common in patients after bipolar coagulation. The limitation of this study is that, in addition to radiological evaluation, microbiological evaluation may be required for EF verification. Apart from this, when the patients were examined clinically, the risk of reoperation was significantly reduced. The fact that patients' pain does not recur is essential in terms of patient satisfaction. Opening of the adhesions formed due to epidural fibrosis in clinically symptomatic patients causes patients to be operated on again. This results in decreased patient comfort, increased surgical complications, and increased hospital costs.

## Conclusions

The complication of EF that develops after lumbar microsurgery operations both impairs patient comfort and brings with it the complications of reoperation. After performing hemostasis with bipolar, coagulating the annulus in the same way may effectively reduce EF and prevent reoperation.
